# Differential stability of therapeutic peptides with different proteolytic cleavage sites in blood, plasma and serum

**DOI:** 10.1371/journal.pone.0178943

**Published:** 2017-06-02

**Authors:** Roland Böttger, Ralf Hoffmann, Daniel Knappe

**Affiliations:** 1 Institute of Bioanalytical Chemistry, Faculty of Chemistry and Mineralogy, Universität Leipzig, Leipzig, Germany; 2 Center for Biotechnology and Biomedicine, Universität Leipzig, Leipzig, Germany; Institut d'Investigacions Biomediques de Barcelona, SPAIN

## Abstract

Proteolytic degradation of peptide-based drugs is often considered as major weakness limiting systemic therapeutic applications. Therefore, huge efforts are typically devoted to stabilize sequences against proteases present in serum or plasma, obtained as supernatants after complete blood coagulation or centrifugation of blood supplemented with anticoagulants, respectively. Plasma and serum are reproducibly obtained from animals and humans allowing consistent for clinical analyses and research applications. However, the spectrum of active or activated proteases appears to vary depending on the activation of proteases and cofactors during coagulation (serum) or inhibition of such enzymes by anticoagulants (plasma), such as EDTA (metallo- and Ca^2+^-dependent proteases) and heparin (e.g. thrombin, factor Xa). Here, we studied the presumed effects on peptide degradation by taking blood via cardiac puncture of CD-1 mice using a syringe containing a peptide solution. Due to absence of coagulation activators (e.g. glass surfaces and damaged cells), visible blood clotting was prevented allowing to study peptide degradation for one hour. The remaining peptide was quantified and the degradation products were identified using mass spectrometry. When the degradation rates (half-life times) were compared to serum derived freshly from the same animal and commercial serum and plasma samples, peptides of three different families showed indeed considerably different stabilities. Generally, peptides were faster degraded in serum than in plasma, but surprisingly all peptides were more stable in fresh blood and the order of degradation rates among the peptides varied among the six different incubation experiments. This indicates, that proteolytic degradation of peptide-based therapeutics may often be misleading stimulating efforts to stabilize peptides at degradation sites relevant only *in vitro*, i.e., for serum or plasma stability assays, but of lower importance *in vivo*.

## Introduction

Proteases are ubiquitous constituents of cells, tissues and body fluids, essential for digestion of food (extracellular), post-translational processing and subcellular localization [1]. Peptide-based drugs are constantly exposed to a wide range of proteases with diverse specificities *in vivo*. Therefore, peptide sequences of potent drug candidates are typically substituted to eliminate motifs recognized by proteases or to incorporate structural features preventing proteolysis [2–6]. The high content of specific and nonspecific proteases in the digestive tract prevents also oral administration in most cases requiring peptide drugs to be typically injected intravenous, intramuscular, subcutaneous, or intraperitoneal [7]. Current efforts to optimize drugs rely frequently on evaluating large numbers of lead compounds and their derivatives, which requires suitable *in vitro* techniques providing reliable predictions of *in vivo* performance and reducing the number of animal experiments.

Peptides administered by the injection routes mentioned above will reach the bloodstream containing proteases participating in hemostasis, fibrinolysis, and tissue conversion, i.e., processes preeminently important in case of injury. Hemostasis, as the first step of wound healing, can be separated in two stages. The primary (cellular) stage constriction of the blood vessel relies on adhesion of a platelet plug at the injured site. In the secondary (plasmatic) stage a thrombus consisting of an aggregated fibrin mesh formed by a complex proteolytic cascade that involves a number of coagulation factors [8]. This process is initiated intrinsically by contact to negatively charged surfaces (e.g., collagen, lipids, bacteria or glass) or extrinsically by exposure of the membrane-bound tissue factor at the injured subendothelium to blood. Activation of two different enzyme cascades trigger the formation of activated thrombin, a protease capable of converting fibrinogen to fibrin. The coagulation factors circulate in blood as inactive zymogens and additionally hemostatic inhibitors, such as antithrombin and heparin, are present preventing undesired clot formation. Activation and inhibition of the clotting cascade requires a permanent balance to maintain a suitable blood flow [9,10].

Collected blood is routinely transformed into plasma or serum depending on the desired analysis. Both terms describe the liquid fraction of blood separated from cellular components. Plasma is produced by adding anticoagulants, such as EDTA or heparin, to prevent blood clotting, while serum is the supernatant obtained after blood coagulation, which is particularly accelerated in the presence of silica particles. Therefore, coagulation factors are not activated in plasma but present in serum, as they are only partly consumed or separated with fibrin clots [11]. This most likely results in different protease compositions and proteolytic activities among blood, serum, and plasma besides different metabolite profiles [12].

In blood coagulation, mostly calcium- and vitamin K-dependent serine proteases are involved (e. g., thrombin) with trypsin-like proteolysis, i.e., cleavage sites C-terminal to lysine or arginine residues in P1 position of certain sequence motifs (S1 Fig) [13]. These proteases can also degrade therapeutic peptides that commonly contain basic residues important for binding to the target via ionic interactions [14,15] or cell entry in eukaryotic and prokaryotic cells [16–18].

In the present study, blood was collected by cardiac puncture directly in a syringe containing a peptide studied for its proteolytic degradation in order to reduce coagulation triggering effects as much as possible. Peptide stabilities were evaluated in blood *ex vivo*, serum obtained from the same animal, and commercial serum and plasma after an incubation period of one hour. It is clearly demonstrated that peptides with short half-life times in commercial serum were more stable in freshly obtained blood. The degradation correlated well to the amounts of metabolites formed by degradation of murine fibrinopeptide A allowing to monitor onset and progression of coagulation.

## Materials and methods

### Peptide synthesis

Peptides were synthesized as C-terminal amides or acids on Rink amide MBHA or Wang resins, respectively, using 9-fluorenylmethoxycarbonyl/*tert*-butyl (Fmoc/^*t*^Bu) strategy as described before [3,19,20], and cleaved with trifluoroacetic acid (TFA) containing a scavenger mixture (12.5% v/v; 1,2-ethandithiole, *m*-cresol, thioanisole, and water; 1/2/2/2, v/v/v/v) for two hours. Peptides were precipitated with cold diethyl ether, dried, and purified by reversed-phase chromatography (RPC) on an Äkta Purifier 10 (GE Healthcare Europe GmbH, Freiburg, Germany) using a linear gradient of aqueous acetonitrile in the presence of TFA (0.1%, v/v) as ion pair reagent on a Jupiter C_18_-column (inner diameter: 21 mm, length: 250 mm, particle size: 15 μm, pore size: 30 nm, Phenomenex Inc., Torrance, CA, USA). A System Gold^®^ 125NM high performance liquid chromatography (HPLC) system (Beckman Coulter GmbH, Krefeld, Germany) was used to evaluate peptide purities utilizing a Jupiter C_18_-column (inner diameter: 2 mm, length: 150 mm, particle size: 5 μm, pore size: 30 nm, column temperature: 60°C; Phenomenex) and a detection wavelength of 214 nm. Monoisotopic masses were determined by matrix-assisted laser desorption/ionization time-of-flight mass spectrometry (MALDI-TOF/TOF-MS; 4700 proteomic analyzer; Applied Biosystems GmbH, Darmstadt, Germany) using α-cyano-4-hydroxycinnamic acid (4 g/L in 60% v/v aqueous acetonitrile containing 0.1% v/v TFA) as matrix.

### Animals

This study was carried out in strict accordance with the recommendations in the Guide for the Care and Use of Laboratory Animals and the protocol was approved by the Animal Care and Usage Committee of the state agency Leipzig (Landesdirektion Leipzig, approval number 24–9162.11/10/77). Female outbred CD-1 mice (Swiss; 14–16 weeks, 28–40 g; Janvier Labs, Saint Berthevin Cedex, France) were acclimatized for at least 7 days. Mice were housed in an IVC system (Ebeco, Castrop-Rauxel, Germany) under specific pathogen-free conditions and water given *ad libitum* and had free access to standard food (R/M-H, ssniff Spezialdiäten GmbH, Soest, Germany). Mice were euthanized by carbon dioxide inhalation and terminal bleeding via cardiac puncture through the skin.

### Sample collection

Aqueous peptide solutions (3 g/L) were filled in a sterile syringe (1 mL, Dispomed Witt oHG, Gelnhausen, Deutschland) through the front end, the stainless-steel needle was attached, and blood (200 to 300 μL) was collected via cardiac puncture to obtain a peptide concentration of approximately 31,5 μmol/L. The syringe was carefully disconnected from the needle and sealed while keeping the needle in the heart. A second empty syringe was attached to the needle and 0.5 to 0.8 mL blood were collected. In parallel, the sealed syringe was gently shaken for one minute to mix the peptide solution with blood and further incubated on a Stuart Rotator SB3 (Barloworld, Scientific LTD, Stone, Staffordshire, U.K.) placed in an incubator at 37°C. Aliquots (0.1 mL) were transferred after 1 and 60 min (1, 10 and 30 min for Api88) into BD Microtainer^®^ tubes with dipotassium ethylenediaminetetraacetic acid (K_2_EDTA; Becton, Dickinson and Company, Franklin Lakes, NJ, USA), gently mixed (1 min), and centrifuged (12,000 x g, 2 min). The plasma sample designated as “blood” (B) was precipitated with trichloroacetic acid (TCA), as described below. Blood collected in the second syringe was transferred to a polypropylene tube (Eppendorf AG, Hamburg, Germany) and a BD Microtainer^®^ serum separator tube (SST; Becton, Dickinson and Company). Polypropylene tubes were centrifuged immediately (12,000 x g, 2 to 4 min) to obtain “direct serum” (DS) as supernatant, whereas the serum separator tubes were incubated (room temperature, 1 h), centrifuged (12,000 x g, 2 min), and collected as “activated serum” (AS). Pooled mouse serum was purchased from PAA Laboratories GmbH (Pasching, Austria) and pooled CD-1 mouse plasma containing K_2_EDTA or lithium heparin were obtained from Innovative Research (Novi, MI, USA).

### Stability assay

Peptides were mixed with serum or plasma to a final concentration of 31.5 μmol/L and incubated (37°C, 750 rpm; Thermomixer, Eppendorf AG, Hamburg, Germany). Aliquots (20 or 40 μL) taken after 0 and 60 min (0, 10 and 30 min for Api88) were mixed with TCA to obtain a final concentration of 3% (w/v) and incubated on ice (10 min). After centrifugation (12,000 x g, 5 min), the supernatant was neutralized with sodium hydroxide (1 mol/L) and stored at −20°C. Samples were analyzed by RP-HPLC on a Jupiter C_18_-column (internal diameter: 2 mm, length: 150 mm, particle size: 5 μm, pore size: 30 nm) using a linear aqueous acetonitrile gradient containing TFA (0.1%, v/v) as ion pair reagent. Peptides were detected by recording the absorbance at 214 nm and quantified by their peak areas relative to the initial peak areas (0 min). All stability tests were performed at least in triplicates.

Metabolites were identified on an Agilent 1100 LC system (Agilent Technologies, Santa Clara, CA, USA) coupled on-line via a UV-detector (absorbance recorded at 214 nm) to the electrospray ionization source on an ion trap mass spectrometer (ESI-IT-MS, Esquire HCT, Bruker Daltonics GmbH, Bremen, Germany). Samples were separated on an Aqua C_18_-column (inner diameter: 2 mm, length: 150 mm, particle size: 3 μm, pore size: 12.5 nm, Phenomenex; column temperature: 60°C) at a flow rate of 0.2 mL/min using a linear gradient of aqueous acetonitrile in the presence of formic acid (FA; 0.1%, v/v) as ion pair reagent. AAYR-Onc112 and LVPR-Onc112 and their corresponding metabolites were quantified by their peak areas (UV) relative to the initial peak areas (0 min). The ESI-IT-MS was operated in positive ion mode. Ionization was carried out at 365°C (source temperature) using nitrogen as curtain gas (40 psi) and dry gas (9 L/min). Mass spectra were acquired for an *m/z* range from 400 to 1300 at a scan rate of 8100 *m/z*-units per second in standard enhanced mode.

## Results

### Blood sample preparations

**Fig 1:**
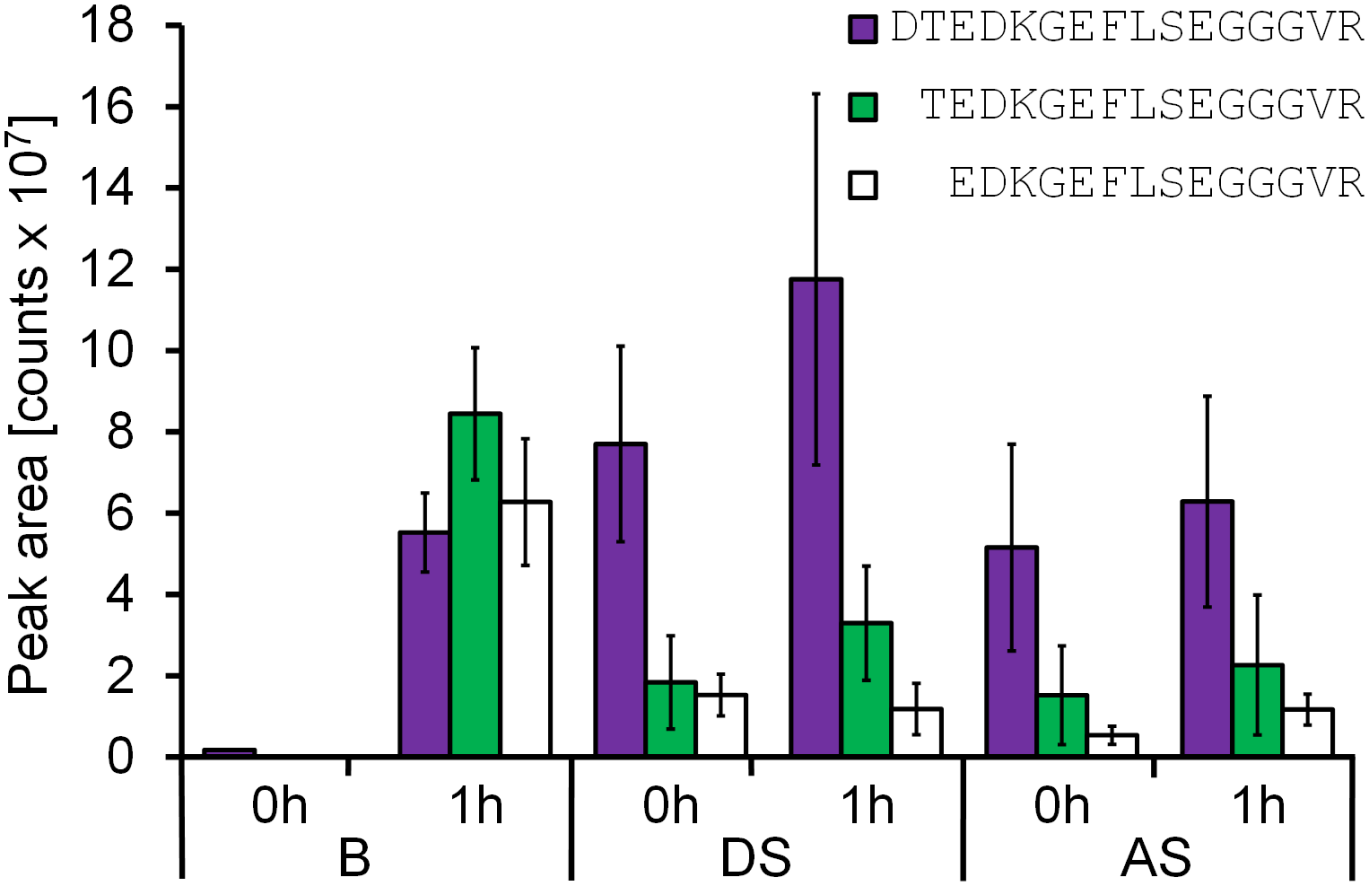
Fibrinopeptide A and its degradation products. Peak areas obtained from extracted ion chromatograms of triply protonated murine fibrinopeptide A (DTEDKGEFLSEGGGVR, purple; *m/z* 565.9) and its triply protonated degradation products TEDKGEFLSEGGGVR (green; *m/z* 527.6) and EDKGEFLSEGGGVR (white; *m/z* 493.9). Six to nine samples were analyzed for each matrix (blood, B; direct serum, DS; activated serum, AS) and time point.

When peptide solutions placed into a syringe were mixed with blood freshly taken via cardiac puncture from mice, coagulation was observed only in one out of 24 syringes after incubating the samples at 37°C for one hour. From all other syringes, liquid blood could be transferred dropwise without visible coagulum into centrifugation tubes containing EDTA. The samples, designated as “blood” (B), were centrifuged and peptides and their degradation products were analyzed. Blood collected afterwards from each mouse in polypropylene tubes immediately started to coagulate and yielded a clear supernatant after 2 min of centrifugation (“direct serum”, DS), although some samples showed a sticky surface most likely formed by fibrinogen that prevented proper pipetting. Thus, centrifugation had to be repeated for half of the samples. The coagulation process in the serum separator tubes SST^™^, accelerated by silica particles, was completed after one hour. After centrifugation, a clear and easy-to-pipet supernatant was obtained (“activated serum”, AS). Although the activation of the coagulation enzymes was not measured, the activity presumably increases from blood to direct serum and further to activated serum. Activation of the coagulation cascade was followed indirectly by analyzing metabolites of murine fibrinopeptide A (FPA) that is released during cleavage of fibrinogen by thrombin. Full length FPA (DTEDKGEFLSEGGGVR) and its two N-terminally truncated metabolites FPA(2–16) and FPA(3–16) were analyzed in six to nine samples of each of the three matrices. Among the six blood samples incubated with peptide for one minute before proteases were inhibited with EDTA, FPA was detected only in one sample at a very low level (B 0h; Fig 1). After an incubation period of one hour, all three peptides were detectable (B 1h) at similar levels indicating that the coagulation process started during incubation without visible clotting. In all serum samples, FPA was the most intense signal corresponding to the intensities obtained in blood after one hour incubation (B 1h), while the intensities of the metabolites were only around 25%. The direct serum samples showed an early onset of coagulation during centrifugation to remove cells and platelets in the absence of EDTA (DS 0h). After an incubation period of one hour, the amount of FPA increased slightly indicating a fully activated thrombin in sample DS 1h. Although not significant, FPA quantities in the activated serum samples (AS 0h and AS 1h) were slightly lower possibly indicating a consumption of fibrinogen and nearly completed coagulation.

### Apidaecin derivatives

**Fig 2:**
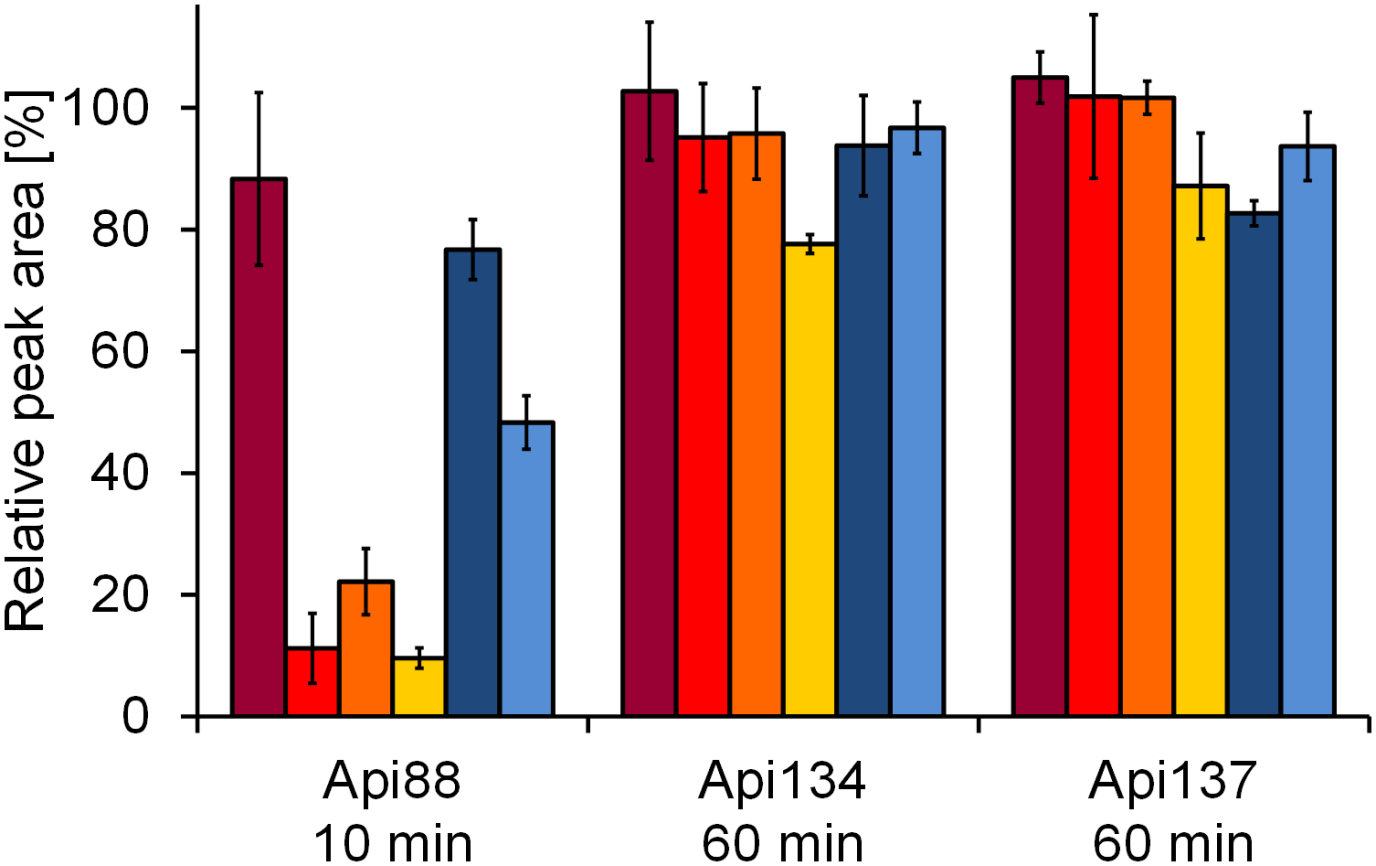
Degradation of apidaecin derivatives. Peptides in blood (dark red), direct serum (red), activated serum (orange), commercial serum (yellow), heparin plasma (dark blue), and EDTA plasma (blue) were analyzed after 10 min (Api88) and one hour (Api134 and Api137) incubation. Separation was performed by RP-HPLC in the presence of 0.1% trifluoroacetic acid and detected by absorbance at 214 nm. Peptide amounts were calculated relative to the quantities determined at time point zero.

Based on apidaecin 1b, a proline-rich antimicrobial peptide isolated from honeybees [21], several analogs were designed and optimized in recent years [19,22,23]. Apidaecin analog Api88 was reported to have a very low *in vitro* serum stability with a half-life time of less than 5 min in mouse serum, whereas further studies increased the half-life times to 4 and 6 h for Api134 and Api137, respectively, by modifying the C-terminal part [19,22]. Compared to Api88 with its C-terminal amide, the more stable peptides contained an Arg17Orn substitution (Api134) and a C-terminal acid (Api137), respectively (Table 1). As expected from an earlier study, 78 ± 2% and 87 ± 9% of the initially added Api134 and Api137 quantities were still present in commercial mouse serum after an incubation period of one hour (Fig 2) [22]. Api134 was stable in all other five matrices (≥94% intact peptide). Api137 was degraded in heparin plasma (83 ± 2% intact peptide) as fast as in commercial serum, while it was stable in the other four matrices (≥94%). It has to be noted, that in most blood samples the intact (stable) peptides were detected with quantities above 100%, which might be related to improper mixing during the incubation period of 1 min, influence of blood cells and platelets or matrix effects, i.e., coeluting substances increasing the signal intensities. Possibly, a peptide concentration gradient is formed in the syringe during blood collection that is not fully balanced over the short 1 minute incubation. However, assuming the most stable peptides, e.g. Api137, as a control for 100% stability, a maximum error of 10% (increase of peptide amount) seems to be acceptable. Due to the reportedly low serum stability of Api88, it was already quantified after 10 min. Expectedly, only 11 ± 6%, 22 ± 5%, and 10 ± 2% of the original peptide amounts were present in direct, activated, and commercial serum, respectively, which is in agreement with the reported degradation rate [22]. Surprisingly, the recovery rates in blood, heparin plasma, and EDTA plasma were 88 ± 14%, 77 ± 5%, and 48 ± 4%, respectively, indicating a half-life time of around 1 hour in fresh mouse blood, which is 30-fold higher than reported for serum. While Api88 was fully degraded in all serum samples after 30 min, 30 ± 14% remained in blood, which was lower than estimated by the 12% loss within the first 10 min. The faster degradation between 10 and 30 min might indicate that the coagulation process started accelerating also degradation of Api88. This assumption is further supported by the relatively high peptide levels detected after 30 min in heparin plasma (36 ± 1%) and EDTA plasma (5 ± 3%), indicating that inhibition of proteases triggering coagulation also reduce degradation of Api88. Mass spectrometry identified Api88(1–17) as major metabolite indicating a tryptic cleavage site between Arg17 and Leu18. This metabolite corresponded to more than 1% of the initial Api88 quantity in all six samples, as judged by the peak areas (absorbance at 214 nm) (Table 2). The peptide amounts found for the metabolite corresponded to the degradation rate of Api88 (data not shown). A second metabolite was Api88(1–16), which was detected slightly above 1% in commercial serum and only by liquid chromatography coupled online to mass spectrometry (LC-MS) in blood, direct serum, and activated serum. Although Orn17 considerably stabilized Api134, it was still degraded in commercial serum yielding Api134(1–17) and (1–16) detectable by LC-MS. More dominant metabolites were Api134(7–18) and Api134 (8–18) with peak areas of around 3% relative to initially added Api134 (Table 2). Degradation of Api134 was very low providing only weak signals of Api134(1–17), Api134(1–15), and Api134(7–18) in activated serum and Api134(7–18) in heparin plasma.

**Table 1:** Sequences and monoisotopic masses of all studied peptides.

Name	Peptide sequence^a^	Monoisotopic mass [Da]		Reference
		calculated	measured	
**Api88**	gu-ONNRPVYIPRPRPPHPRL-NH_2_	2289.3	2289.3	[19]
**Api134**	gu-ONNRPVYIPRPRPPHPOL-NH_2_	2247.3	2247.3	[22]
**Api137**	gu-ONNRPVYIPRPRPPHPRL-OH	2290.3	2290.2	[22]
**Onc18**	VDKPPYLPRPRPPRRIYNR-NH_2_	2388.4	2388.3	[3]
**Onc72**	VDKPPYLPRPRPPROIYNO-NH_2_	2304.3	2304.3	[3]
**Onc112**	VDKPPYLPRPRPPRrIYNr-NH_2_	2388.4	2388.3	[4]
**AAYR-Onc112**	AAYRVDKPPYLPRPRPPRrIYNr-NH_2_	2849.6	2850.6	[20]
**LVPR-Onc112**	LVPRVDKPPYLPRPRPPRrIYNr-NH_2_	2853.7	2854.6	[20]

^a^ gu, O, and r denote *N*,*N*,*N’*,*N’*-tetramethylguanidino, l-ornithine and d-arginine, respectively.

**Table 2:** Sequences of all degradation products identified in blood, serum, and plasma samples.

Precursor	Metabolite	Metabolite peptide sequence^a^	Matrix^b^	Monoisotopic mass [Da]	
				Calc.	Meas.
**Api88**	1–17	gu-ONNRPVYIPRPRPPHPR-OH	All	2177.2	2177.1
	1–16	gu-ONNRPVYIPRPRPPHP-OH	CS	2021.1	2021.1
**Api134**	7–18	YIPRPRPPHPOL-NH_2_	CS	1454.8	1454.8
	8–18	IPRPRPPHPOL-NH_2_	CS	1291.8	1291.8
**Api137**	1–17	gu-ONNRPVYIPRPRPPHPR-OH	VB, DS, CS	2177.2	2177.1
	1–16	gu-ONNRPVYIPRPRPPHP-OH	CS	2021.1	2021.1
**Onc18**	1–18	VDKPPYLPRPRPPRRIYN-OH	B, DS, AS, CS	2233.1	2233,3
	1–17	VDKPPYLPRPRPPRRIY-OH	B, DS, AS, CS	2119.2	1119.2
	1–15	VDKPPYLPRPRPPRR-OH	EP	1842.9	1843.1
	1–14	VDKPPYLPRPRPPR-OH	All	1687.0	1687.0
	2–14	DKPPYLPRPRPPR-OH	HP	1588.0	1587.9
**Onc72**	1–18	VDKPPYLPRPRPPROIYN-OH	All	2191.2	2191.4
	1–15	VDKPPYLPRPRPPRO-OH	CS, HP, EP	1801.0	1800.9
	1–14	VDKPPYLPRPRPPR-OH	All	1687.0	1687.0
**AAYR-Onc112**	3–23	YRVDKPPYLPRPRPPRrIYNr-NH_2_	All	2707.5	2707.5
	4–23	RVDKPPYLPRPRPPRrIYNr-NH_2_	All	2544.5	2544.6
	5–23 [Onc112]	VDKPPYLPRPRPPRrIYNr-NH_2_	All	2388.4	2388.6
**LVPR-Onc112**	4–23	RVDKPPYLPRPRPPRrIYNr-NH_2_	All	2544.5	2544.4
	5–23 [Onc112]	VDKPPYLPRPRPPRrIYNr-NH_2_	All	2388.4	2388.3

Monoisotopic masses of all degradation products were identified using LC-MS. Only metabolites with peak areas corresponding to at least 1% of the peak area of the initially incubated peptide quantities were taken into account.

^a^ gu, O, and r denote *N*,*N*,*N’*,*N’*-tetramethylguanidino, l-ornithine and d-arginine, respectively.

^b^ Metabolites were identified in blood (B), direct serum (DS), activated serum (AS), commercial serum (CS), EDTA plasma (EP), and heparin plasma (HP). “All” denotes that this metabolite was detected in all six matrices.

Api137 was cleaved in commercial serum (Fig 2) between His15 and Leu18 with Api137(1–17) and Api137(1–16) detected at intensities corresponding to ~3% of the original quantity of Api137, whereas Api137(1–15) was only detected by LC-MS. These metabolites were detected at even lower amounts in direct and activated serum. Degradation in heparin plasma was comparable to commercial serum yielding four metabolites identified in LC-MS: Api137(1–17), Api137(1–16), Api137(1–15), and Api137(7–18), which were also detected in the blood sample despite the low degradation rate of Api137. Api137(1–17) was even detected at the 4% level (UV-signal).

### Oncocin derivatives

**Fig 3:**
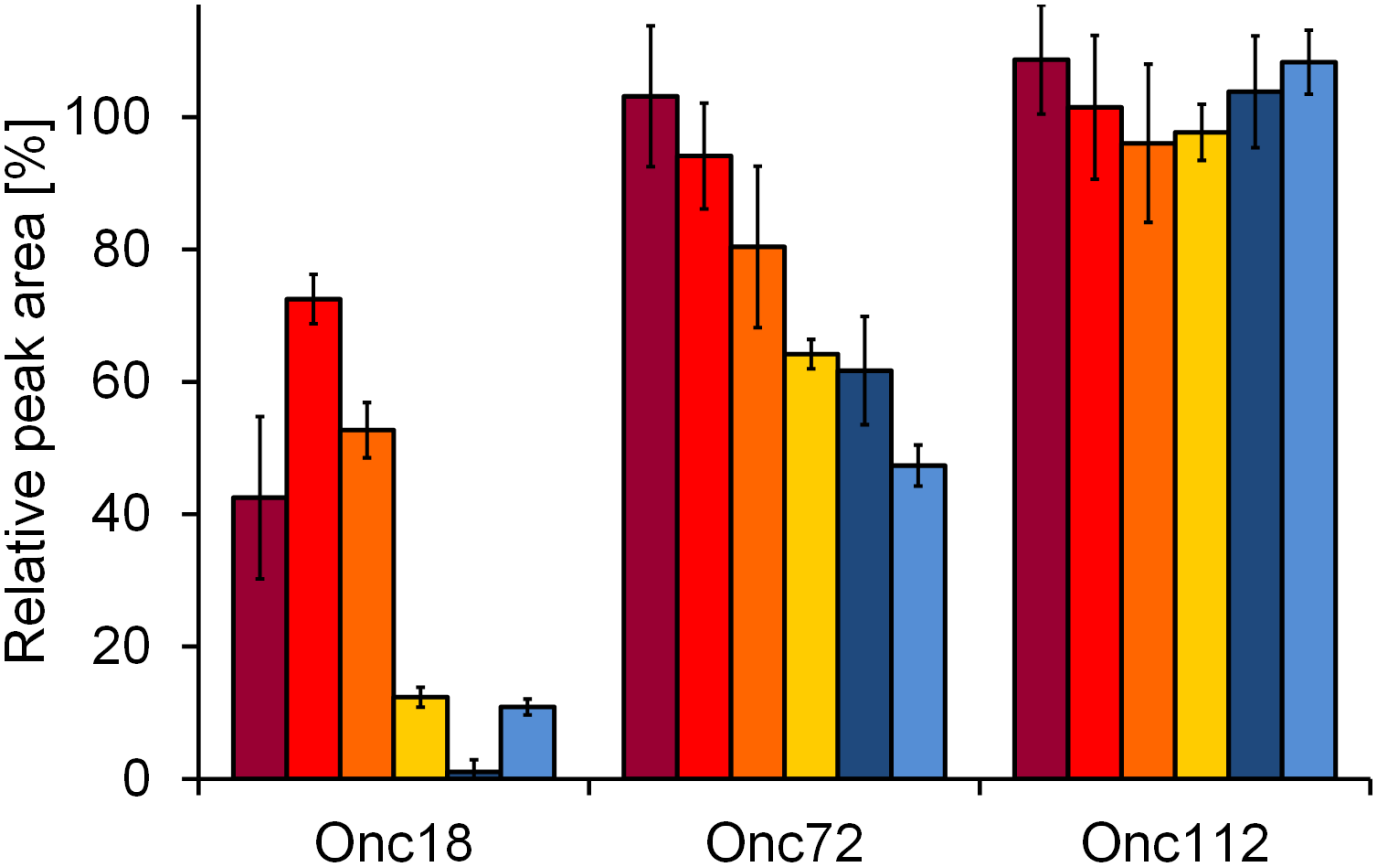
Degradation of oncocin derivatives. Onc18, Onc72, and Onc112 were analyzed after one hour incubation in blood (dark red), direct serum (red), activated serum (orange), commercial serum (yellow), heparin plasma (dark blue), and EDTA plasma (blue). Peptides were separated by RP-HPLC in the presence of 0.1% trifluoroacetic acid and detected by absorbance at 214 nm. Peptide amounts were calculated relative to the quantities determined at time point zero.

Oncocin derivative Onc18, which is an optimized analog of *Oncopeltus* antibacterial peptide 4 (Table 1), has a reported serum half-life time of 20 min with around a quarter of the peptide remaining after an incubation period of 1 h [3,24]. Here, around two-fold less intact peptide was observed in commercial serum (12 ± 1%; Fig 3). Dominant cleavage sites were between Arg14-Arg15 and Asn18-Arg19. In earlier studies, substitution of positions 15 and 19 to l-ornithine (Orn) or d-arginine (d-Arg) yielded the stabilized analogs Onc72 and Onc112, respectively (Table 2) [3,4], with serum half-life times of 3 h and more than 8 h, respectively, in mouse serum. The high stability of Onc112 was confirmed here with less than 2% being degraded after 1 hour in commercial serum (Fig 3) and at least 96% remaining in all other matrices. Onc72 was degraded fast in commercial serum, i.e., 64 ± 2% remaining after one hour compared to 80% in a previous study [3]. Similar data were obtained for heparin (62 ± 8%) and EDTA plasma (47 ± 3%). Onc72 was more stable in activated (80 ± 12%) and direct serum (94 ± 8%), whereas it was not degraded in blood (103 ± 11%). As expected, activation of the coagulation process appeared to slowly increase the degradation of Onc72, but surprisingly proteolytic activity of both plasma samples was comparable to commercial serum. Thus, metabolite Onc72(1–15), only found in commercial serum and plasma (Table 2), could indicate the presence of additional or “late-activated” proteases, that are not necessarily part of the coagulation. Although, Onc72 was mostly degraded C-terminal to Arg14 and Asn18, an additional cleavage between Orn15 and Ile16 could explain its unexpectedly fast degradation in plasma.

Onc18 was the least stable oncocin analog with less than 13% detected in commercial serum and both plasma samples after 1 hour (Fig 3). Surprisingly and in contrast to Api88, it was four- to seven-fold more stable in blood (42 ± 12%), direct (72 ± 4%), and activated serum (53 ± 4%) with Arg14-Arg15 as dominant cleavage site in all samples. The signal corresponding to metabolite Onc18(1–14) was especially intense in plasma (S2 Fig) with only one more metabolite detected in heparin plasma, i.e., Onc18(2–14), and one more in EDTA plasma, i.e., Onc18(1–15). Blood and serum samples showed comparable metabolite patterns, but in addition to Onc18(1–14) metabolites Onc18(1–17) and Onc18(1–18) were also detected, most likely produced by consecutive C-terminal exopeptidase cleavages (Table 2 and S2 Fig).

### Linker-Onc112 peptides

**Fig 4:**
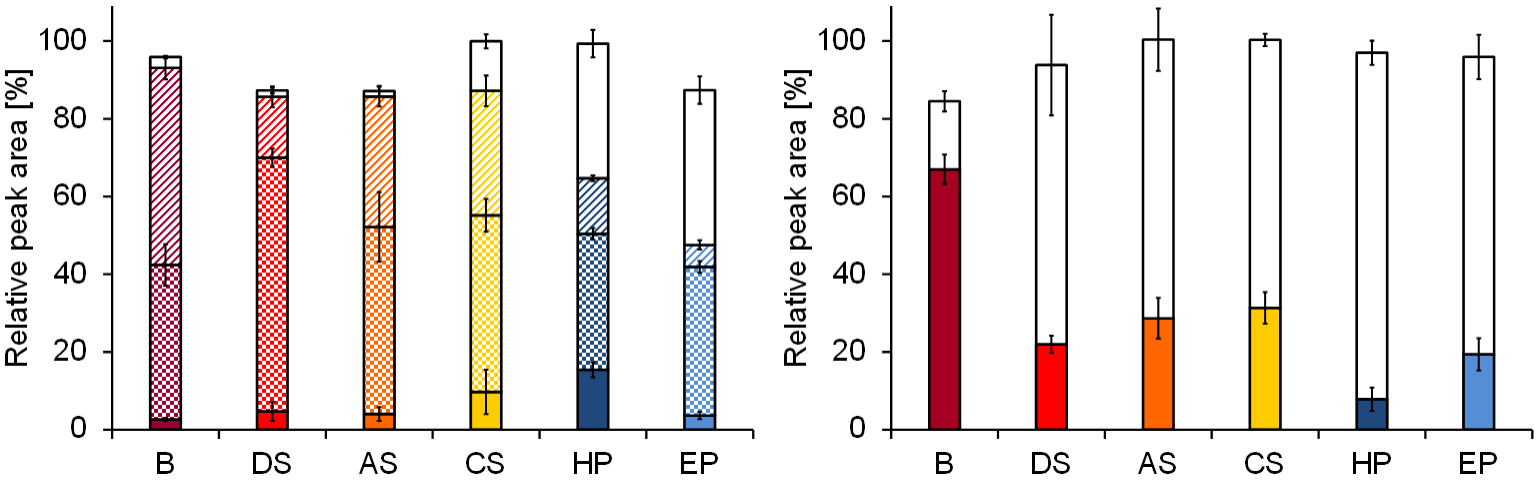
Degradation of elongated Onc112 derivatives. AAYR-Onc112 (left) and LVPR-Onc112 (right) were analyzed after one hour incubation in blood (dark red), direct serum (red), activated serum (orange), commercial serum (yellow), heparin plasma (dark blue), and EDTA plasma (blue). Peptides were separated by RP-HPLC in the presence of 0.1% formic acid and detected by absorbance at 214 nm. Peptide amounts were calculated relative to the quantities determined at time point zero. Onc112 (white) was released from both constructs, whereas metabolites YR-Onc112 (squared) and R-Onc112 (striped) were detected only for AAYR-Onc112.

In a recent study, Onc112 was elongated by a linker sequence to facilitate its release from PEGylated prodrugs by serum proteases, such as AAYR-Onc112 and LVPR-Onc112 that released 85% and 57% of Onc112 when incubated in 25% aqueous commercial serum [20]. Assuming a three-fold faster degradation in full serum [3], only 5% and 14% of the prodrugs, respectively, should be present, which is around two times less than detected here (Fig 4). The other matrices showed similar release rates for AAYR-Onc112 with 3 ± 1 to 15 ± 2% of the intact peptide remaining. As expected, degradation of LVPR-Onc112 was slower and 8 ± 3 to 31 ± 4% intact peptide were found in serum and plasma samples. Blood was again different with 67 ± 4% of LVPR-Onc112 left after one hour, i.e., three-fold more than in the other matrices. For this peptide, the release of Onc112 nicely correlated to degradation of its prodrug with both peptide quantities resembling almost 100% of the initial amounts in all matrices except for blood (85%; Fig 4). The peptide quantities missing in blood could be attributed to metabolite LVPR-Onc112(4–23), which was also detected by LC-MS in all other samples at low intensities, although the background prevented a precise quantification. Cleavage within the linker sequence AAYR was already reported before [20] and obtained here after both Val2 and Pro3 (Table 2). These non-desired metabolites were obtained at different quantities and partially co-eluted as indicated by UV-RP-HPLC using trifluoroacetic acid as ion pair reagent. Separation was improved in the presence of formic acid and suitable for quantification. In both plasma samples, similar amounts of Onc112 were released (HP: 35 ± 4% and EP: 40 ± 4%), but only at half quantities in commercial serum (13 ± 2%) and very small amounts in blood, direct serum, and activated serum (<3%). The remaining peptide was degraded to undesired cleavage products that together with the quantities of Onc112 represented the initial amounts of AAYR-Onc112 (Fig 4). Degradation products AAYR-Onc112(3–23) and AAYR-Onc112(4–23) were present at different ratios of 4:3 in blood, 2:3 in activated and commercial serum, 1:3 in EDTA plasma, 1:7 in heparin plasma HP, and 1:4 in direct serum indicating different protease compositions among the matrices.

## Discussion

Peptides represent promising therapeutic lead structures due to their high efficacy and selectivity, large range of target proteins, and low tissue accumulation [7]. Additionally, they provide a wide chemical and biological diversity, can be identified at both peptide and nucleic acid levels, and are easy and fast to synthesize at reasonable costs. Peptides are degraded by proteases that are universally present in all organisms and the environment providing fast degradation rates and thus preventing long-term accumulations with potentially toxic effects, as common to many small molecules including marketed antibiotics. However, this inherent advantage often prevents therapeutic applications demanding long circulation times and ideally oral administration. Thus, administration typically relies on injection or infusion to enable circulation of therapeutic peptides in the blood stream besides topical applications. In all cases, proteases immediately start to degrade the peptides before they reach the respective target. The resulting short and typically hydrophilic peptides are cleared effectively by renal excretion leading to usually fast declining levels. Different strategies can improve their stability by incorporation of non-canonical or unnatural amino acids, the corresponding d-enantiomers or backbone modifications [3–6,17,22,25,26]. Proteolytic degradation in blood is typically studied *in vitro* by incubating the peptides and their stabilized analogs in serum or plasma, which allows also the identification of cleavage sites and their iterative stabilization by the above-mentioned strategies.

Indeed, the half-life times of oncocin derivative Onc18 in mouse serum increased from 25 min to 3 h (Onc72) and even more than 8 h (Onc112) when substituting both Arg15 and Arg19 by ornithine or d-arginine, respectively. As expected from the different *in vitro* stability, Onc112 exhibited an improved pharmacokinetic profile and around fivefold higher plasma levels than Onc72 after intravenous application in mice [27]. Surprisingly, a pharmacokinetic study performed in parallel for apidaecin 1b analogs Api88 and Api137 revealed similar plasma levels of both peptides [23] despite drastically different half-life times of only 5 min and 6 h, respectively, in mouse serum [22]. The data presented here nicely resolve this apparent discrepancy. When incubated in blood at 37°C, 88 ± 15% of Api88 was detected after 10 min and still 30 ± 14% after 30 min, whereas Api137 was only slightly degraded during 60 min. As the serum stabilities of both peptides are still different from the pharmacokinetics, it is tempting to speculate that Api88 is degraded by a protease activated during coagulation, as indicated by the initially slow degradation that increases in the second period. Indeed, Pro and Arg at positions P2 and P1 represent a preferable cleavage site of activated thrombin in mammalian species [28]. The difference in the specificity of thrombin to Api88 and Api137 relates most likely to the C-terminus, i.e., amide versus free acid of Leu19 at P1´ of thrombin, respectively. The free acid of Api137 might have a similar effect as thrombin disfavors aspartic and glutamic acids in position P2´. Similarly, Api134 with its free acid was equally stable as Api137 with ornithine at P1 most likely preventing cleavage by thrombin as well. Thus, all three peptides appear to have similar stabilities in blood only influenced by the low amounts of thrombin present to balance the permanent activation and inactivation of the coagulation cascade [9,10]. We confirmed this assumption by comparing the proteolytic cleavage of Api88 and Api137 by human α-thrombin, which possesses the same Pro-Arg substrate specificity as the murine homolog [28]. While Api88 was already completely degraded to Api88(1–17) after one hour, Api137 remained mostly intact with only low amounts of metabolite Api137(1–17) released after 24 h (S3 Fig and S1 Text).

In contrast, Onc18 was preferably cleaved between Arg14 and Arg15 in commercial serum, EDTA plasma, and heparin plasma leading to lower stabilities compared to blood, direct serum, and activated serum. According to the MEROPS database for proteolytic enzymes [29], Pro-Arg-Arg on positions P2-P1-P1´ enable cleavage by thrombin and other proteases possibly indicating the complex and diverse compositions of blood, serum, and plasma. However, substituting arginine by ornithine or d-arginine improved stabilities of Onc72 and Onc112 in all matrices.

The most stable oncocin analog Onc112 was recently used to evaluate linker sequences for their release kinetics in commercial serum to design PEGylated prodrugs [20]. Here, Onc112 was released from LVPR-Onc112 considerably more slowly in blood than in the other matrices. Its release from AAYR-Onc112 was similarly slow in blood and sera, but faster in plasma. In contrast, the precursor AAYR-Onc112 was degraded in all matrices at similar rates. This seemingly contradiction might indicate two competing cleavage sites, which were degraded preferably in blood and sera. In addition, thrombin cleaved expectedly C-terminal to Arg4 in LVPR-Onc112, as LVPR is used as tag to cleave recombinant fusion proteins by thrombin [30], but the hydrolysis was slower than for PHPR-L in Api88 (S3 Fig). Interestingly, the degradation of LVPR-Onc112 was faster in heparin plasma than in EDTA plasma, whereas Api88 was faster cleaved in EDTA plasma. Thus, the activities of thrombin and other proteases possibly depend on the plasma type. Indeed, previous reports using recalcified citrated plasma showed that EDTA incompletely inhibits reactivation of thrombin (0.39 U/mL), but heparin does (0.06 U/mL) [31]. Although speculative, slightly faster degradation of LVPR-Onc112 could rely on a second protease inhibited by EDTA but not by heparin, possibly independent of the coagulation process.

Considering the sequential and overall stabilities of all eight peptides, five different patterns of matrix stability can be proposed (Table 3) with the general conclusion that peptides appear to be usually most stable in fresh blood and least stable in serum. This correlates to earlier studies profiling proteolytic activities using internally quenched fluorogenic probes [32,33]. Different metabolic markers resulting from thrombin assisted protein degradation, e.g., FPA and its metabolites, provide some information about the coagulation progress and the proteolytic activity in serum and plasma [34,35]. Indeed, FPA was absent in blood immediately after sample collection and the quantities of FPA, FPA(2–16), and FPA(3–16) were different after one hour than in serum prepared accordingly. However, the analysis of peptide stability in fresh blood as best predictor of *in vivo* studies, is limited to short incubation times up to one hour and will most likely not become a routine assay. Therefore, peptide degradation should be analyzed in serum and plasma and compared with the degradation profile obtained in fresh blood samples during the first hour. This could allow together with a database search (e.g. MEROPS database) for major proteolytic cleavage sites to adjust the half-life times obtained in plasma or serum assays to predict peptide degradation in the blood stream more precisely for a given sequence.

**Table 3:** Stability of eight peptides in fresh blood, serum, and plasma.

Peptides	Sequential stability	Overall stability
**Api134 / Api137 / Onc112**	Blood = Plasma = Serum	High
**Onc18 / Onc72**	Blood = Serum > Plasma	Medium
**Api88**	Blood > Plasma > Serum	Low
**AAYR-Onc112**	Blood = Plasma = Serum	Low
**LVPR-Onc112**	Blood > Serum > Plasma	Low

## Conclusion

Proteolytic stabilities of therapeutic peptides vary among serum, plasma, and fresh blood for each peptide depending on the liquid portion of blood derived after coagulation or addition of coagulation inhibitors. The intact peptide amount found after incubation in freshly derived blood in a syringe differs considerably from at least one of the other two matrices. As fresh blood, preferably murine blood for preclinical studies, is usually not available on a laboratory bench for high-throughput assays, peptides should be tested in both commercially available serum and plasma. Interpretation of the data should rely on database searches for potential cleavage sites attributed to common protease families (e.g. MEROPS database). It was demonstrated that arginine residues especially prone to protease cleavage can be favorably substituted by non-proteinogenic amino acids ornithine and d-arginine to stabilize peptides in blood, serum, and plasma. This well-known approach should be complimented with by pharmacokinetic studies of potential therapeutic peptides in early development stages. As renal clearance overlays the blood stability *in vivo*, only identification and quantification of all major metabolites would allow judging the stability of the peptide *in vivo*.

## Supporting information

S1 Fig Schematic peptide-protease-binding complex. Peptide side chains P located in the corresponding protease binding pockets S (modified after Karstad et al. [13]).
(TIF)

S2 Fig RP-HPLC of Onc18 and its metabolites after incubation in different matrices. Chromatograms recorded after incubating Onc18 in different matrices for one hour (UV absorbance). Labeled signals indicate metabolites identified by ESI-IT-MS, whereas no metabolites were identified in the fractions corresponding to unlabeled peaks. Onc18 incubated in heparin plasma coeluted with Onc18(1–14) at 19.4 min in the presence of 0.1% formic acid. Quantification of Onc18 (Fig 2) relied therefore on the peak areas obtained by RP-HPLC using trifluoroacetic acid (0.1% v/v) as ion pair reagent.
(TIF)

S3 Fig Stability of Api88, Api137, and LVPR-Onc112 against human α-thrombin. Chromatograms recorded after incubation of Api88 (a), Api137 (b), or LVPR-Onc112 (c) with human α-Thrombin for 0, 1, and 24 hours (UV absorbance). Separation was achieved in the presence of 0.1% formic acid and metabolites were identified by on-line by mass spectrometry.
(TIF)

S1 Text Method α-thrombin stability assay. (DOCX)
