# Supplementary figures and images for: Differential stability of therapeutic peptides with different proteolytic cleavage sites in blood, plasma and serum
Source: PLoS One. 2017 Jun 2;12(6):e0178943. doi: 10.1371/journal.pone.0178943 (PMC5456363; doi:10.1371/journal.pone.0178943)

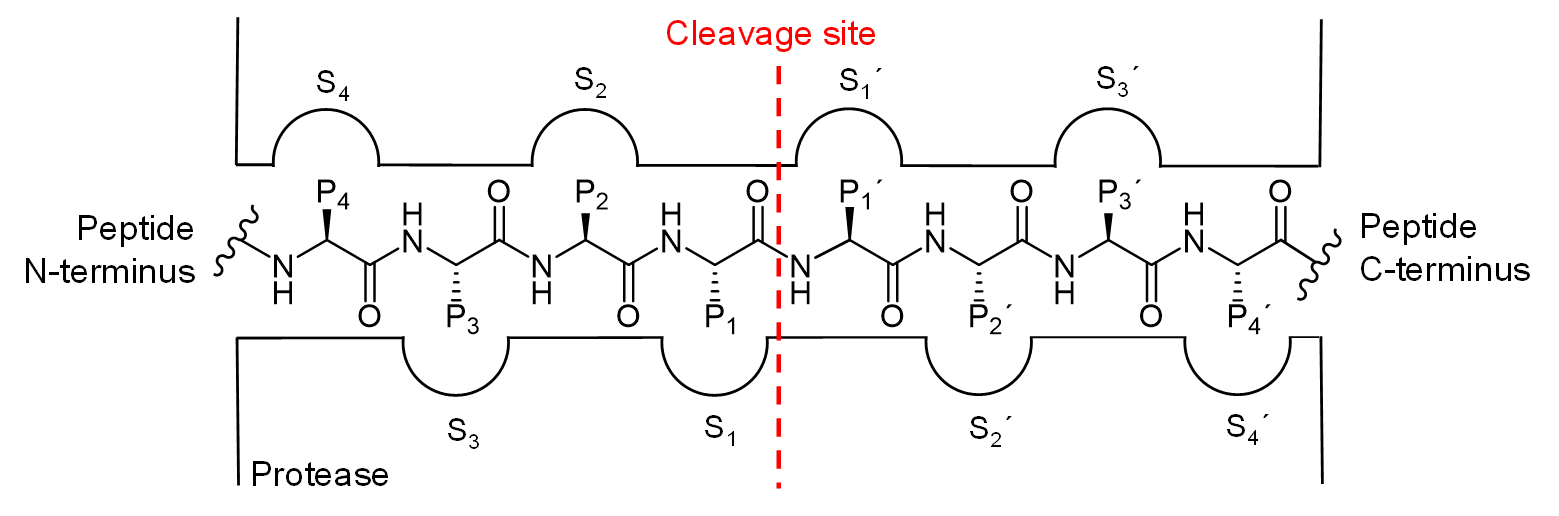

Supplement: S1 Fig — Peptide side chains P located in the corresponding protease binding pockets S (modified after Karstad et al. [13]). (TIF) [file pone.0178943.s001.tif]
