# Supplementary material for: Differential stability of therapeutic peptides with different proteolytic cleavage sites in blood, plasma and serum
Source: PLoS One. 2017 Jun 2;12(6):e0178943. doi: 10.1371/journal.pone.0178943 (PMC5456363; doi:10.1371/journal.pone.0178943)
Supplement: S1 Text — (DOCX) [file pone.0178943.s004.docx]

**S1 Text. Method α-Thrombin stability assay.**

Peptides (3 g/L) were mixed with human α-thrombin (0.1 U/µL; 0.75 µL; Hematologic Technologies Inc., Essex Junction, VT, USA) in buffer (1 mmol/L, 30 µL) containing 20 mmol/L Tris-buffered saline (pH 8.4), 150 mmol/L sodium chloride, and 2.5 mmol/L calcium chloride and incubated (37°C, 750 rpm; Thermomixer, Eppendorf AG). Aliquots (8 µL) taken after 0, 1 and 24 h were mixed with phenylmethylsulfonyl fluoride (PMSF) to a final concentration of 1 mmol/L to inhibit α-thrombin. Samples were analyzed on an Agilent 1100 LC system (Agilent Technologies) coupled on-line via a UV-detector (absorbance recorded at 214 nm) to the electrospray ionization source of an ion trap mass spectrometer (Esquire HCT, Bruker Daltonics GmbH). Separation was achieved on an Aqua C_18_-column (inner diameter: 2 mm, length: 150 mm, particle size: 3 µm, pore size: 12.5 nm, Phenomenex; column temperature: 60°C) at a flow rate of 0.2 mL/min using a linear gradient of aqueous acetonitrile in the presence of 0.1% (v/v) formic acid) as ion pair reagent. The ESI-IT-MS was operated in positive ion mode. Ionization was carried out at 365°C (source temperature) using nitrogen as curtain gas (40 psi) and dry gas (9 L/min). Mass spectra were acquired for a *m/z* range from 400 to 1000 at a scan rate of 8100 *m/z*-units per second in standard enhanced mode.
